# Study on wettability of water stemming for blasting dust adjusted by surfactants and inorganic salts

**DOI:** 10.1098/rsos.241250

**Published:** 2025-01-08

**Authors:** Fengjie Chen, Zejun Zhou, Ying Liang, Xiaowen Liu, Xiaoguang Wang, Pu Wang, Bolei Chen, Yong Liang, Yawei Wang

**Affiliations:** ^1^ State Key Laboratory of Precision Blasting, Jianghan University, Wuhan 430056, People’s Republic of China; ^2^ State Key Laboratory of Environmental Chemistry and Eco-toxicology, Research Center for Eco-environmental Sciences, Chinese Academy of Sciences, Beijing 100085, People’s Republic of China

**Keywords:** blasting dust, water stemming, surfactants, inorganic salts, wettability

## Abstract

Water stemming is an efficient method of removing blasting dust by wetting. There is still a lack of methods for rapid optimization of water stemming components with high wettability. Herein, blasting dust was collected from a tunnel in Chongqing (China) to investigate its removal performance by different water stemmings. The two most important components of blasting dust were SiO_2_ and CaCO_3_ by characterization analysis. Notably, hydrophilic blasting dust has significantly more SiO_2_ than hydrophobic blasting dust. The density functional theory calculation predicted the wettability of water stemming containing sucrose fatty acid ester (SE) higher than that of water stemming containing other surfactants. Moreover, the water contact angle and surface tension experiments determined the addition of inorganic salts to the water stemming containing SE could increase its wettability, with the addition of Al^3+^ giving the best performance. The sink test and water retention experiment further prove that our synthesized water stemming has a good wetting ability on both hydrophobic and hydrophilic blasting dust. The findings of this study advance the development of reliable methods for optimizing water stemming with high wettability for removing the blasting dust.

## 1. Introduction

The drilling and blasting method is the most commonly used method for constructing a tunnel, which greatly promotes economic development [1,2]. However, under the influence of blasting, a significant amount of dust will be produced in this process. The operator’s work and movement in such an environment will cause the basting dust to be inhaled into the human body and then through the respiratory tract into the lungs, resulting in the serious occupational disease pneumoconiosis [3–5]. Thus, it is significant to strengthen the control technology of blasting dust in the tunnel construction process.

At present, blasting dust control technology such as water pre-wet dust removal [6], water curtain dust removal [7], ventilation dust removal [8], water mist dust removal [9] and water pressure blasting dust removal [10] can reduce the amount of dust. In particular, water pressure blasting dust removal technology has received considerable attention due to its simple operation, low cost, no additional energy input, suitability for various scenarios and high dust removal efficiency [11–14]. The essence of water pressure blasting dust removal is that droplets with a certain speed and particle size are produced through the water stemming during blasting, and then wrap the dust-containing air generated by blasting. Finally, dust removal is achieved through the interaction between fog drops and dust [15,16]. Although ordinary water stemming can reduce the production of blasting dust to a certain extent, its result of reducing dust is not ideal [17]. One of the reasons is that the surface tension of water is large and the wettability is small [18]. Another reason is that different dust has different physical and chemical properties, resulting in differences in the interaction between water stemming and blasting dust [19,20].

Changing the component of the water stemming can enhance its trapping capacity and wettability, resulting in a high removal efficiency of blasting dust [21]. Early studies reported that the addition of surfactants to the water stemming can reduce the surface tension, which can improve its atomization effect during the blasting process and then increase the trapping efficiency of fog droplets on blasting dust [22,23]. Besides, the addition of inorganic salts such as calcium chloride, magnesium chloride, etc. to water stemming can increase the wettability, which can promote the settlement of blasting dust [24]. However, the concentration of each component and the interference between each component in the water stemming will affect its trapping capacity and wettability. Meanwhile, the physical and chemical properties of blasting dust will affect the interaction between it and each component of the water stemming [25–27]. Considering the discussion above, we thought changing the surface tension and wettability of water and clarifying the physical and chemical properties of blasting dust could improve the dust removal performance of the water stemming from the water pressure blasting dust removal process.

Herein, blasting dust was collected from a tunnel in Chongqing (China) and used to investigate its removal performance by different components water stemming. The physical and chemical properties of blasting dust were characterized by scanning electron microscope (SEM), X-ray diffraction (XRD) and X-ray photoelectron spectroscopy (XPS). Different species and concentrations of surfactants and inorganic salts were chosen to construct the water stemming. The interaction between blasting dust and water stemming was investigated by density functional theory (DFT) calculations, surface tension and contact angle analyses, which ultimately led to the efficient removal of blasting dust. This work establishes a strategy for selecting water stemming to remove different types of blasting dust, which provides a certain theoretical support for the development of precision blasting.

## 2. Material and methods

### 2.1. Material

Blasting dust was collected from the blasting site of Yunyang Tunnel in Chongqing. Cationic surfactant cetyl trimethyl ammonium bromide (CTAB, 99%, CAS 57-09-0), anionic surfactant sodium dodecyl sulfate (SDS, 92.5–100.5%, CAS 151-21-3) and non-ionic surfactant sucrose fatty acid ester (SE, HLB-13, CAS 37318-31-3) were purchased from the Shanghai Acmec Biochemical Co., Ltd (Shanghai, China). NaCl was purchased from Sinopharm Chemical Reagent Co., Ltd (Shanghai, China) for analytical purity. CaCl_2_ (96%, CAS 10043-52-4) and AlCl_3_ (99%, CAS 7446-70-0) were purchased from the Shanghai Macklin Biochemical Technology Co., Ltd (Shanghai, China).

### 2.2. Characterization

Hydrophilic and hydrophobic dust were separated from blasting dust through water separation experiments. The morphology and element distribution of the dust samples were acquired using a GeminiSEM 300 (ZEISS, Germany). Additionally, the dust samples were characterized by XRD using an Empyrean multi-function XRD instrument (PANalytical, The Netherlands). The isothermal adsorption–desorption curve, specific surface area and micropore size of the dust were determined using an ASAP 2460 adsorption analyser (Micromeritics, USA). Furthermore, the surface properties of the dust were analysed by K-Alpha XPS, and the contact angle between the dust and the solution was measured using an SZ-CAMB3 contact angle measuring instrument (Sunzern, China).

### 2.3. Density functional theory calculation

All the DFT and *ab initio* molecular dynamics (AIMD) computations were performed using the Cambridge Sequential Total Energy Package based on the pseudopotential plane wave method. Electron–ion interactions were described using the ultrasoft potentials. A plane-wave basis set was employed to expand the wave functions with a cut-off kinetic energy of 450 eV. For the electron–electron exchange and correlation interactions, the functional parametrized by Perdew–Burke–Ernzerhof, a form of the general gradient approximation, was used throughout.

During the geometry optimizations, all the atom positions were allowed to relax. In this work, the Brillouin-zone integrations were conducted using Monkhorst–Pack grids of special points with a separation of 0.08 Å^−1^. The convergence criterion for the electronic self-consistent field loop was set to 2 × 10^−6^ eV atom^−1^. The atomic structures were optimized until the residual forces were below 0.05 eV Å^−1^. The van der Waals interaction was described using the DFT-D2 method proposed by Grimme.

All adsorptive binding energies (*E*_ads_) used in this article were defined as


$$E_{ads}=E_{total}-\left(E_{slab}+E_{adsorbate}\right),$$

where *E*_total_ is the total energy of the slab/adsorbate interacting system and *E*_slab_ and *E*_adsorbate_ are the energies of the slab and the isolated adsorbate, respectively.

### 2.4. Contact angles experiments

The water stemming containing 1.00 mM SE, CTAB and SDS were prepared. Then, the SZ-CAMB3 contact angle measurement instrument is used to detect the contact angle between water stemming and blasting dust.

### 2.5. Surface tension analysis

Surfactant solutions with concentrations of 2.00, 1.00, 0.50, 0.25, 0.10, 0.05 and 0.01 mM were prepared. NaCl, CaCl_2_ and AlCl_3_ were then added to each surfactant solution and the concentration of inorganic salts in the solution was adjusted to 10.00, 5.00, 1.00, 0.50 and 0.10 mM, respectively. Subsequently, the surface tension of the resulting solutions was measured.

The surface tension (γ) of the mixed solutions was determined by the platinum plate method using a BZY-1 automatic surface tension meter with the surfactant concentration variable (*c*). Then, the γ-lgc relationship curve was plotted. The concentration at the inflection point of the curve is the critical micelle concentration (CMC). Finally, the experimental data were analysed to determine the changes in the CMC of the surfactant solution after the addition of different concentrations and types of inorganic salts and to select the optimal water stem composition.

### 2.6. Sink experiment

Quickly introduce 100 mg of hydrophobic or hydrophilic blasting dust into a beaker containing 25 ml of water or water stemming solution. Start timing as soon as the dust makes contact with the liquid surface, and stop when all the dust has completely submerged. If the submersion time exceeds 600 s, the test is discontinued, indicating that the blasting dust is resistant to wetting by the solution.

### 2.7. Water retention experiment

Evenly distribute water or water stemming solution over 100 mg of hydrophilic or hydrophobic blasting dust spread flat in a Petri dish. The samples were weighed using an electronic balance, and the initial mass was recorded as *M*_0_. All samples were then dried in an oven at 90°C, with their mass (*M*_i_) measured every 20 min until a constant weight was achieved. The evaporation rate is calculated using the following formula:


$$\eta =\frac{\left(M_{0}-M_{i}\right)}{AT}.$$

In the equation, *η* represents the dust suppressant evaporation rate; *M*_0_ stands for the mass before evaporation; *M*_i_ denotes the mass after evaporation; *A* represents the evaporative area of the dust sample; and *T* signifies the evaporation time.

## 3. Results and discussion

### 3.1. Physical and chemical properties of the blasting dust

**Figure 1.**
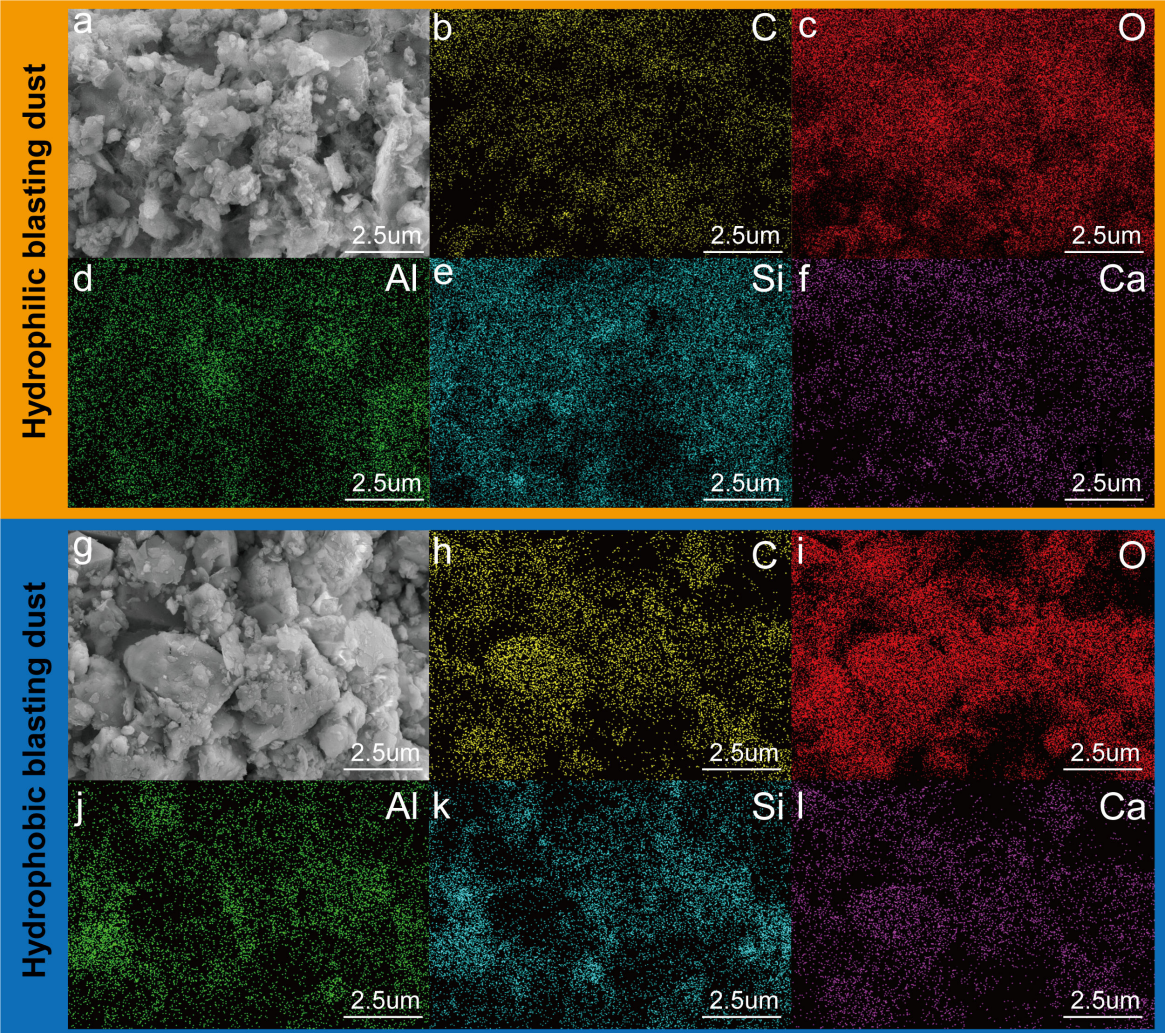
(*a*) FESEM Images of hydrophilic blasting dust; EDX elemental mapping of hydrophilic blasting dust (*b*) C, (*c*) O, (*d*) Al, (*e*) Si and (*f*) Ca; (*g*) FESEM Images of hydrophobic blasting dust; EDX elemental mapping of hydrophobic blasting dust (*h*) C, (*i*) O, (*j*) Al, (*k*) Si and (*l*) Ca.

The hydrophilic and hydrophobic composition of the blasting dust surface has significantly influenced its wettability and removal performance by water stemming [28,29]. As shown in electronic supplementary material, figure S1, the blasting dust was put into a beaker containing deionized water, and separated into two different parts due to its wettability. The blasting dust sample floating on the surface is hydrophobic dust, and the ones deposited on the bottom are hydrophilic dust. XRD was used to understand the difference between hydrophilic and hydrophobic blasting dust. In electronic supplementary material, figure S2, the hydrophilic blasting dust exhibits 11 significant peaks at 20.84°, 23.44°, 26.67°, 29.38°, 36.11°, 39.43°, 43.27°, 48.49°, 50.11°, 57.37° and 59.98° which represented (100), (012), (101), (104), (110), (113), (202), (116), (112), (122) and (211), indicating that the main composition of hydrophilic blasting dust was SiO_2_, CaCO_3_ and Al_2_O_3_. The planes with (100), (101) and (112) of the hydrophobic blasting dust were obviously lower than the hydrophilic blasting dust, indicating the content of SiO_2_ in hydrophobic blasting dust was much lower than the hydrophilic blasting dust. Field emission scanning electron microscope (FESEM) and energy-dispersive X-ray spectroscopy (EDX) were further used to determine the physical properties of the blasting dust. The SEM results showed the blasting dust is mainly blocky and flake, and the particles of hydrophilic blasting dust are significantly higher than those of hydrophobic blasting dust (figure 1*a*,*g*), consisting of the former research. Besides, electronic supplementary material, figure S3 shows that based on IUPAC, both the nitrogen sorption isotherms of hydrophilic and hydrophobic blasting dust belong to the typical type IV isotherms, suggesting that these samples were rich in mesoporous structural characteristics. The Brunauer–Emmett–Teller (BET) surface areas, Barrett–Joyner–Halenda pore diameters and pore volumes were described in electronic supplementary material, table S1. The data showed that the BET surface area of hydrophilic blasting dust was lower than that of hydrophobic blasting dust. This was probably because the particles of hydrophilic blasting dust were higher than those of hydrophobic blasting dust. The higher BET surface can adsorb more air to form the tight air film on the dust surface, which hinders the contact between water and dust. Figure 1*b–f,h*–l and electronic supplementary material, table S2 show that the C, O, Al, Si and Ca elements were evenly distributed in the blasting dust. Notably, the content of C and Ca elements in the hydrophilic blasting dust (12.41 and 19.28%) is lower than that in the hydrophobic blasting dust (14.13 and 28.47%). In comparison, the content of O and Si elements in the hydrophilic blasting dust (49.80 and 13.62%) is higher than that in hydrophobic blasting dust (47.76 and 6.38%). This result further demonstrated that the SiO_2_ component of hydrophilic was higher than that of hydrophobic blasting dust.

**Figure 2.**
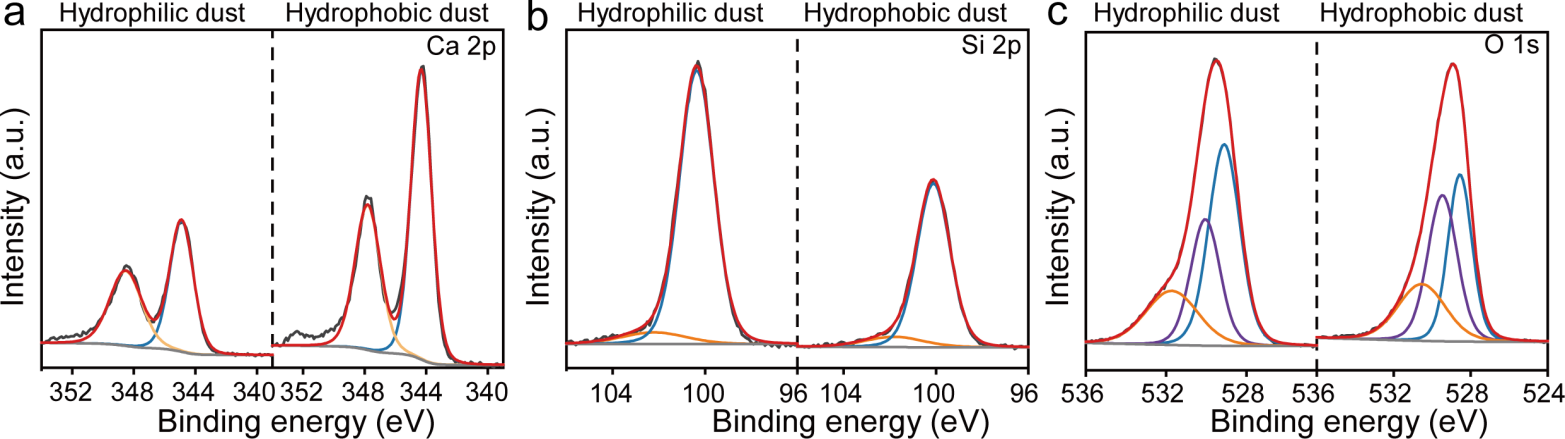
XPS spectra of hydrophilic and hydrophobic blasting dust (*a*) Ca 2*p*, (*b*) Si 2*p* and (*c*) O 1*s*.

The surface functional groups were another factor affecting the hydrophilic and hydrophobic properties of blasting dust [30]. The Ca 2*p* XPS spectra of hydrophilic and hydrophobic blasting dust are shown in figure 2*a*. The result showed that Ca 2*p* has two peaks at 344.9 and 348.5 eV, corresponding to Ca 2*p*_3/2_ and Ca 2*p*_1/2_, thereby confirming the existence of Ca–O bonding in the lattice. In figure 2*b*, Si 2*p* has two peaks at 100.4 and 102.2 eV, corresponding to Si–O–Si and Si–OH. The intensity of Ca 2*p* of hydrophobic blasting dust was higher than that of hydrophilic blasting dust, and the intensity of Ca 2*p* of hydrophobic blasting dust was lower than that of hydrophilic blasting dust. These results further indicated that SiO_2_ and CaCO_3_ were the main components of hydrophilic and hydrophobic blasting dust respectively. Figure 2*c* exhibits three peaks at 529.1, 530.0 and 531.7 eV, which were ascribed to lattice oxygen, –OH and surface adsorption impurities, respectively. It can be seen that the fraction of hydrophilic groups (–OH group) in hydrophilic blasting dust is 17.38% lower than that of hydrophobic blasting dust, which is consistent with the conclusion that the wetting performance of hydrophilic blasting dust is stronger than that of hydrophobic blasting dust [31].

### 3.2. Wettability of water stemming for blasting dust adjusted by surfactant

**Figure 3.**
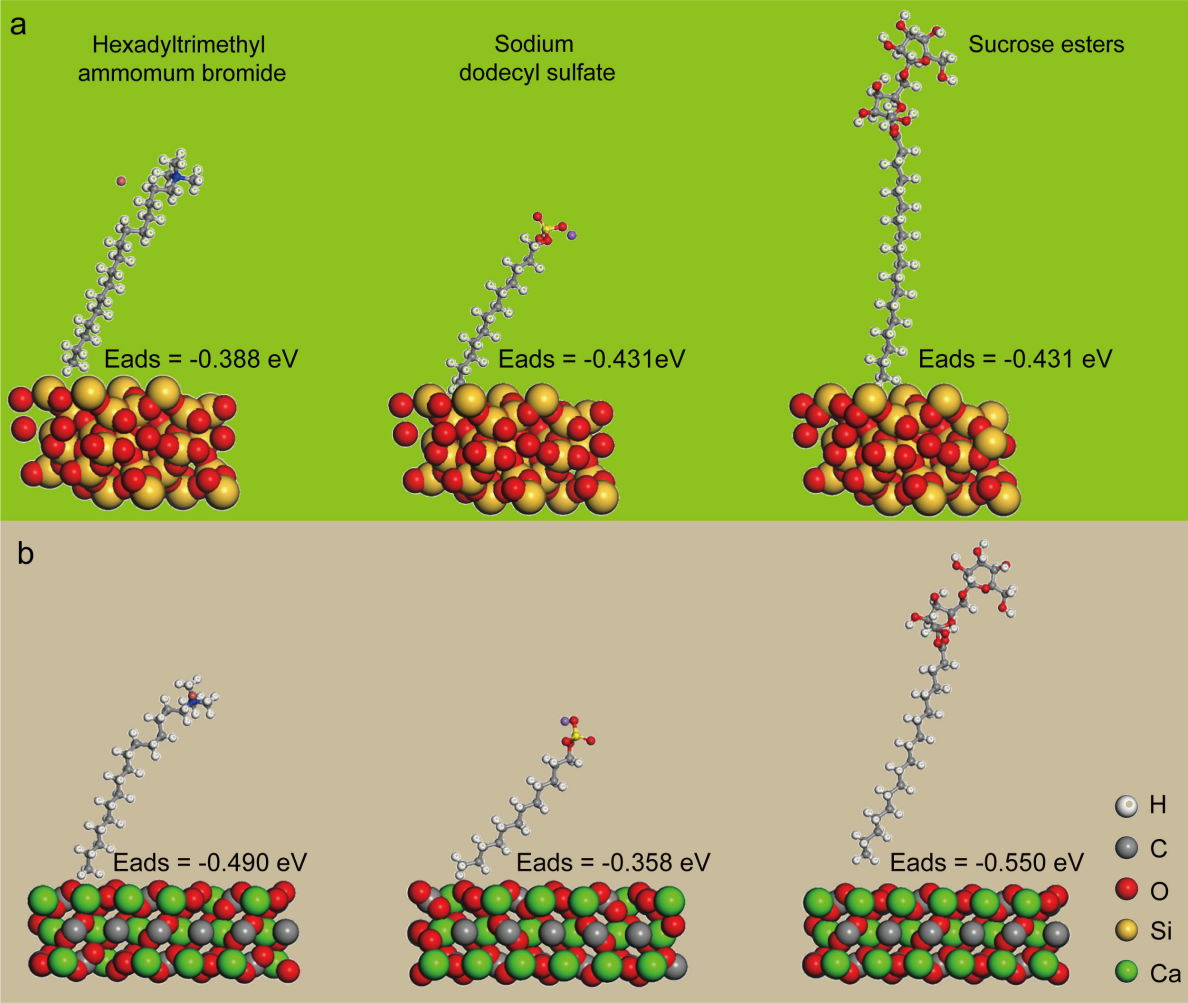
Adsorption configurations and adsorption energies (Eads) of CTAB, SDS and SE on (*a*) SiO_2_ and (*b*) CaCO_3_ surfaces using DFT methodology.

Surfactant is an abbreviation for surface active agent, which can change the water surface property to enhance its wettability [32]. We chose the SDS (anionic surfactant), CTAB (cationic surfactant) and SE (non-ionic surfactant) as the components of the water stemming and investigate its wettability for the hydrophilic and hydrophobic blasting dust. Because the main components of the hydrophilic and hydrophobic blasting dust were the SiO_2_ and CaCO_3_, we calculated the adsorption energies between the surfactant and SiO_2_ or CaCO_3_ to evaluate the wettability of water stemming samples. As shown in figure 3*a*, the result showed that the adsorption energies between SiO_2_ and SDS, CTAB and SE were −0.388, −0.431 and −0.431 eV, respectively, indicating the ability of surfactant to adsorb on the SiO_2_ surface in the order CTAB < SDS = SE. Similarly, the adsorption energies between CaCO_3_ and SDS, CTAB and SE were −0.490, −0.358 and −0.550 eV, respectively, indicating the ability of surfactant to adsorb on the CaCO_3_ surface in the order SDS < CTAB < SE (figure 3*b*). These findings suggest that water containing SE exhibits the strongest interaction with both hydrophilic and hydrophobic blasting dust among all the samples.

**Figure 4.**
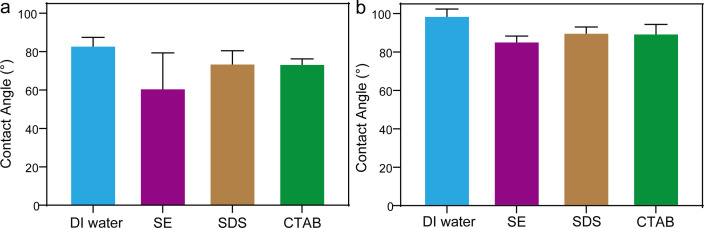
The contact angles of the water stemming containing DI water, SE, SDS and CTAB with (*a*) hydrophilic and (*b*) hydrophobic blasting dust.

The contact angle experiment was conducted to further assess the interaction between the surfactants and blasting dust. In figure 4*a* and electronic supplementary material, figure S4, the contact angle between hydrophilic blasting dust and deionized (DI) water, SE, SDS and CTAB was 81.1°, 40.1°, 67.5° and 71.6°, indicating the wettability of SE for hydrophilic blasting dust was higher than that of DI water, SDS and CTAB. In addition, the contact angle between hydrophobic blasting dust and DI water, SE, SDS and CTAB was 95.4°, 82.3°, 89.9° and 83.2° (figure 4*b* and electronic supplementary material, figure S4), indicating the wettability of SE for hydrophobic blasting dust was higher than that of DI water, SDS and CTAB. These results demonstrate that water containing SE had superior wetting performance for both hydrophilic and hydrophobic blasting dust compared with SDS and CTAB. Additionally, the findings align well with the DFT results, which showed stronger adsorption energies for SE, supporting its better ability to enhance wettability. Therefore, based on the superior wettability observed for blasting dust, the water stemming solution containing SE was chosen for subsequent experiments.

### 3.3. Wettability of water stemming containing sucrose fatty acid ester for blasting dust adjusted by inorganic salt

**Figure 5.**
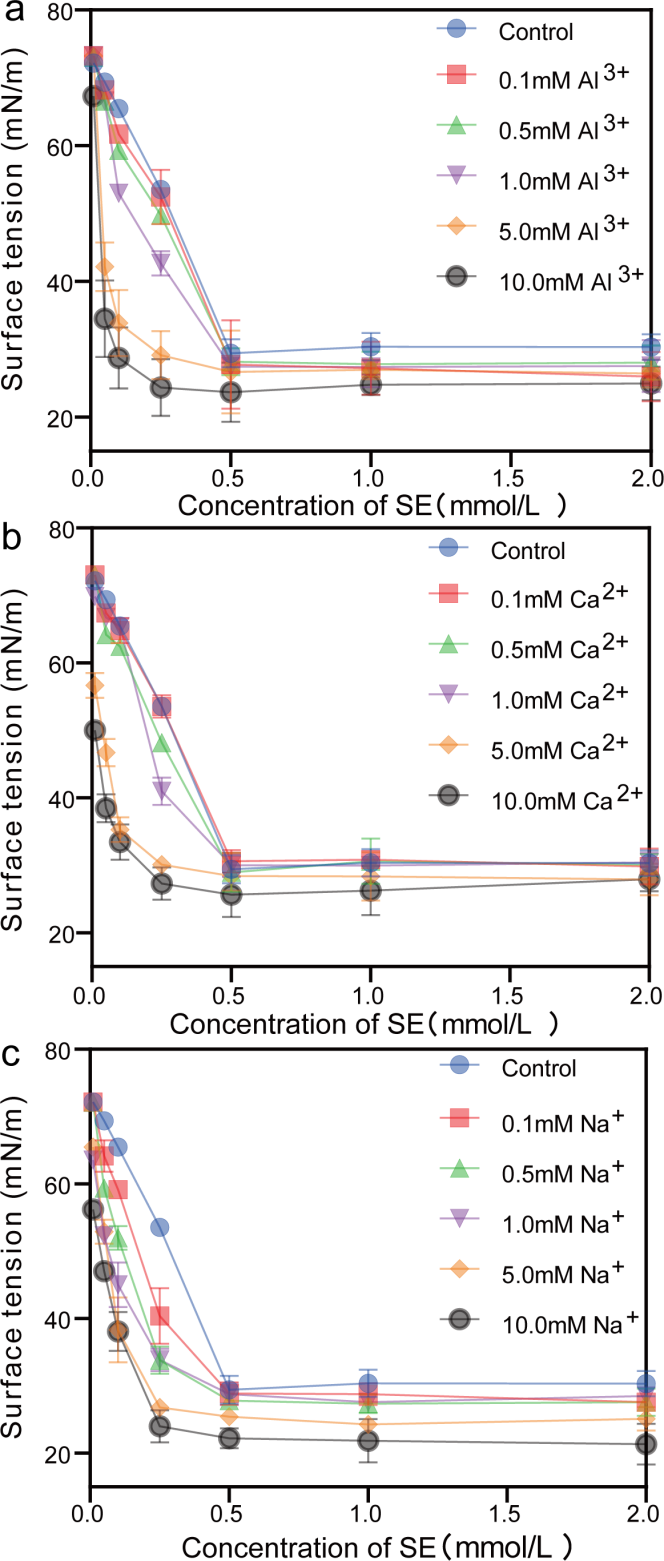
The surface tension of water stemming containing SE and (*a*) Al^3+^, (*b*) Ca^2+^ and (*c*) Na^+^ ions at different concentrations.

**Figure 6.**
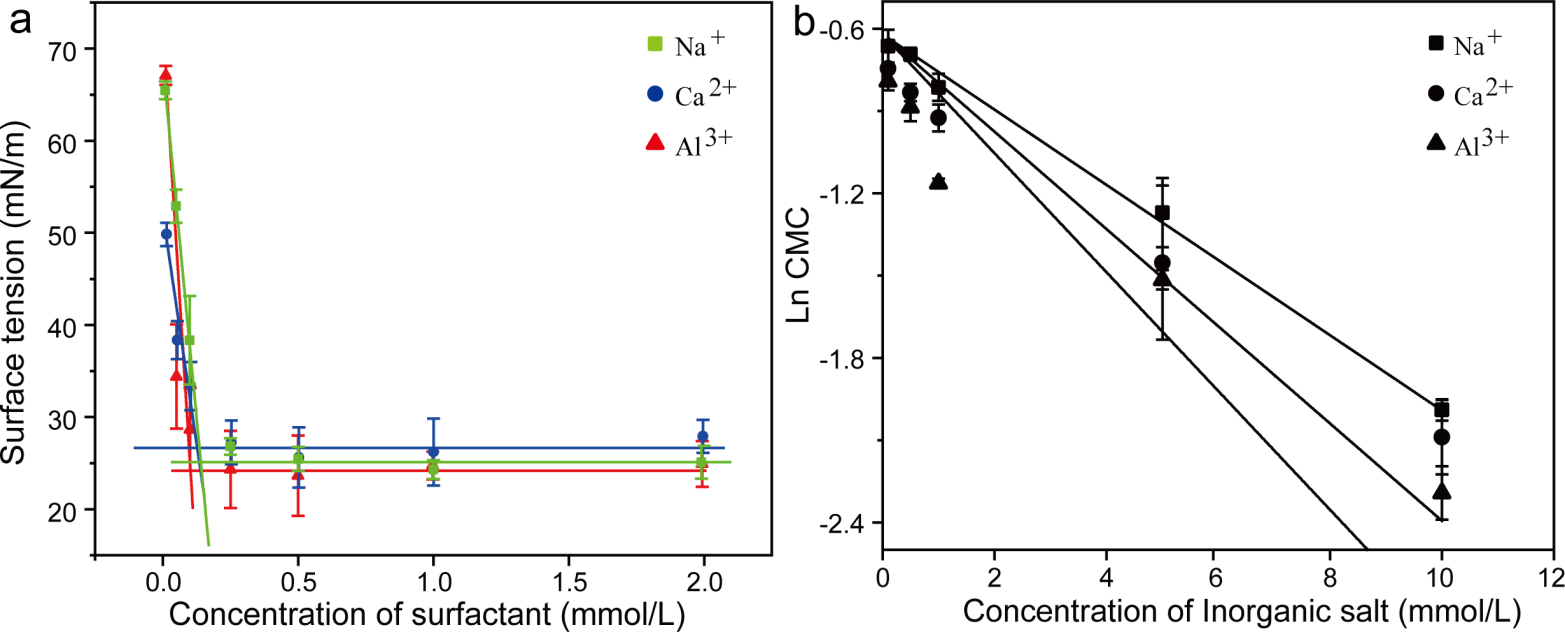
(*a*) The CMC of SE containing 10 mM Al^3+^, Ca^2+^ and Na^+^; (*b*) Plots of the logarithm of the CMC values for SE containing different concentrations of salts.

**Figure 7.**
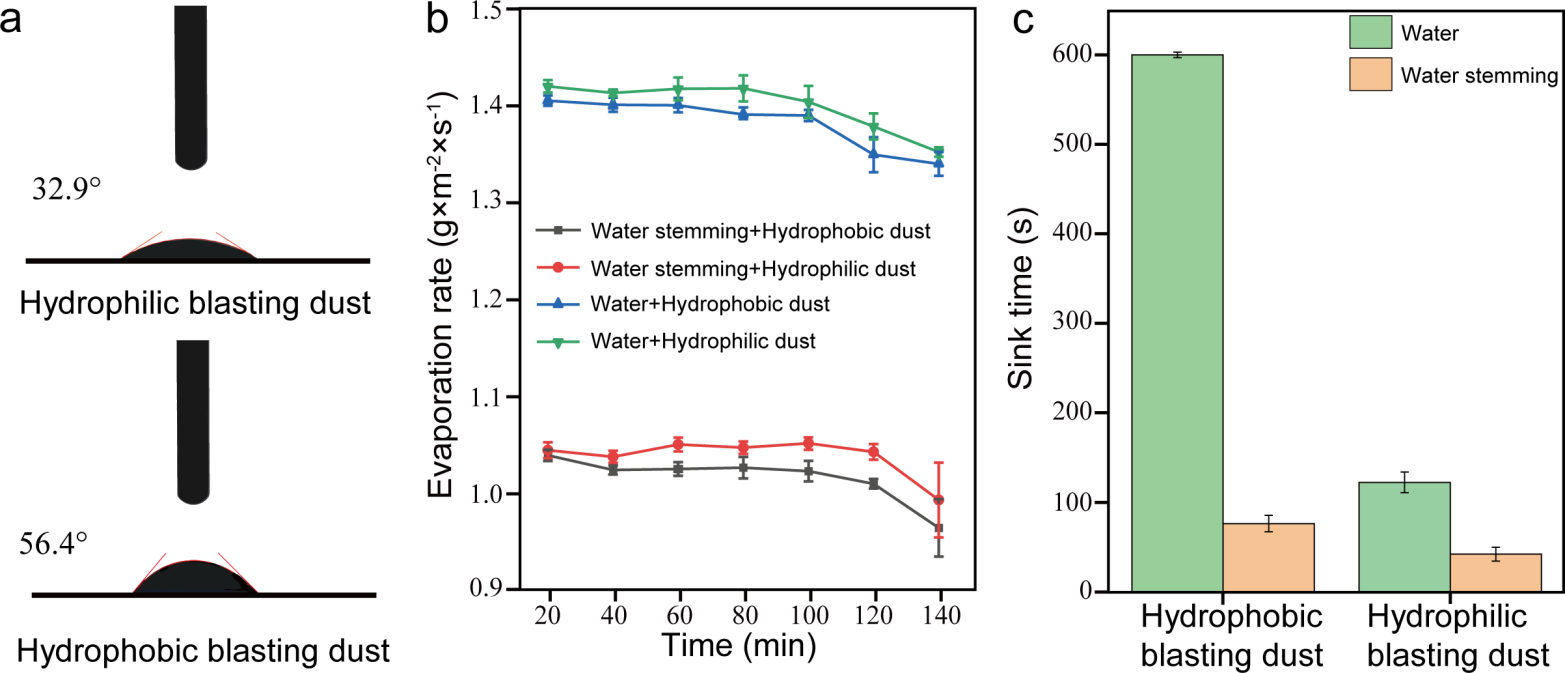
(*a*) The contact angle of hydrophilic and hydrophobic blasting dust with water stemming containing 10 mM Al^3+^ and 0.25 mM SE. (*b*) The evaporation rate of water and water stemming containing 10 mM Al^3+^ and 0.25 mM SE on hydrophilic and hydrophobic blasting dust. (*c*) The sink time of hydrophilic and hydrophobic blasting dust in water and water stemming containing 10 mM Al^3+^ and 0.25 mM SE.

The inorganic salt can significantly affect the equilibrium surfactant behaviour to change its wettability by changing the equilibrium surface tensions and CMC [33,34]. CMC is the lowest concentration at which surfactant molecules form micelles in solution. It is an important parameter that affects the wettability of surfactant solution. We added Na^+^, Ca^2+^ and Al^3+^ in the water stemming containing SE to evaluate its wettability for blasting dust. As shown in figure 5*a*–*c*, the results showed that the surface tension of the water stemming decreased with the increase of SE concentration, then reached a plateau, indicating adding SE in the water stemming can reduce its surface tension. In general, the lower the surface tension of aqueous solution, the stronger its wettability. When adding inorganic salt, the surface tension of the water stemming decreased with the increase of Na^+^, Ca^2+^ and Al^3+^ concentration, indicating that inorganic salt can affect the surfactant behaviour and enhance its wettability. Then, we calculated the CMC under these conditions (figure 6*a*); the results showed that when adding 10 mM Na^+^, Ca^2+^ or Al^3+^, the CMC of the water stemming containing SE was changed from 0.25 to 0.14 mM, 0.12 mM or 0.10 mM, respectively, indicating the influence of inorganic salt on the CMC of the water stemming containing SE in the order Na^+^ < Ca^2+^ < Al^3+^. The effect of inorganic salt on the non-ionic surfactant CMC is accomplished by the ‘salting out’ or ‘salting in’ of the hydrophobic group in the solution [35]. Upon dissolution of inorganic salts in water, the electric interaction between ions and dipoles causes water molecules to aggregate around the ions, leading to a reduction in free water [36]. Consequently, the salting-out effect facilitates the precipitation of non-ionic surfactants from water, thereby resulting in a decrease in CMC [37,38]. The activity coefficient theory proposed by McDevit and Long is frequently employed to discuss and elucidate the salting-out effect of inorganic salts on non-ionic surfactants [39]. Therefore, for the addition of inorganic salt, the change of the non-ionic surfactant is given by the relation


$$In\;\;CMC=In\;\;CMC_{0}\;-\;k_{s}C_{s},$$

where CMC and CMC_0_ represent the CMCs of surfactants under conditions with and without salt, respectively, *k*_s_ is the salt constant, and *C*_s_ is the concentration of the salt. The higher the value of *k*_s_, the more pronounced the salting-out effect becomes, and the lower the CMC of the surfactant [40]. Based on the results of linear fitting, the magnitude of the *k*_s_ value is Al^3+^ > Ca^2+^ > Na^+^ (figure 6*b*). Based on these discussions, the water stemming containing 10 mM Al^3+^ and 0.25 mM SE exhibits the best wettability. Furthermore, the contact angle analysis indicated that when using water stemming containing 10 mM Al^3+^ and 0.25 mM SE, the contact angle between water stemming and hydrophilic and hydrophobic blasting dust was changed from 40.1° and 82.3° to 32.9° and 56.4° (figure 7*a*), respectively, suggesting that this water stemming has good performance to wet the blasting dust.

Water retention and sink time are important indicators for evaluating the dust suppression performance of water stemming [41,42]. As shown in figure 7*b*, evaporation rates during water evaporation from both hydrophilic and hydrophobic blasting dust under different treatment conditions: water-stem containing 10 mM Al^3+^ and 0.25 mM SE-treated sample < water-treated sample, indicating that the water retention performance of blasting dust treated with 10 mM Al^3+^ and 0.25 mM SE is better than that of water-treated blasting dust. Moreover, the sink analysis indicated that when using water stemming containing 10 mM Al^3+^ and 0.25 mM SE, the sink time between water-stem and hydrophilic and hydrophobic blasting dust was changed from 113.95 and 587.86 s to 47.34 and 76.48 s (figure 7*c*), respectively, suggesting that this water-stem has good performance to sink the blasting dust.

## 4. Conclusion

In this study, we have adjusted the components of water stemming and applied them to achieve high wettability for blasting dust. The main components of blasting dust were SiO_2_, CaCO_3_ and Al_2_O_3_ by the characterization analysis. Notably, hydrophilic blasting dust has significantly more SiO_2_ and surface hydroxyl functional groups than hydrophobic blasting dust. Moreover, adding surfactant into the water stemming could promote its wettability for blasting dust by DFT and water contact angle experiments, with the addition of SE giving the best performance. Further preference experiments showed that the addition of inorganic salts to the water stemming containing SE could reduce its surface tension and thus increase its wettability for blasting dust, with the addition of Al^3+^ giving the best performance. Sink test and water retention experiments further proved that our synthetic water stemming has a good wetting ability for blasting dust. In summary, the findings of this study advance the development of reliable methods for water stemming with high wettability.

## Data Availability

The original data for the article is available online [43]. Supplementary material is available online [44].
